# Multi-Position Inertial Alignment Method for Underground Pipelines Using Data Backtracking Based on Single-Axis FOG/MIMU

**DOI:** 10.3390/mi15091168

**Published:** 2024-09-21

**Authors:** Jiachen Liu, Lu Wang, Yutong Zu, Yuanbiao Hu

**Affiliations:** Faculty of Engineering Technology, China University of Geosciences (Beijing), Beijing 100083, China; 2002220039@email.cugb.edu.cn (J.L.); 3002220025@cugb.edu.cn (Y.Z.); hyb@cugb.edu.cn (Y.H.)

**Keywords:** pipeline three-dimensional trajectory measurement, initial alignment, multi-position initial alignment, data backtracking

## Abstract

The inertial measurement method of pipelines utilizes a Micro-Electro-Mechanical Systems Inertial Measurement Unit (MIMU) to get the three-dimensional trajectory of underground pipelines. The initial attitude is significant for the inertial measurement method of pipelines. The traditional method to obtain the initial attitude uses three-axis magnetometers to measure the Earth’s magnetic field. However, the magnetic field in urban underground pipelines is intricate, which leads to the initial attitude being inaccurate. To overcome this challenge, a novel multi-position initial alignment method based on data backtracking for a single-axis FOG and a three-axis Micro-Electro-Mechanical Inertial Measurement Unit (MIMU) is proposed. Firstly, the configuration of the sensors is determined. Secondly, according to the three-point support structure of the pipeline measuring instrument, a three-position alignment scheme is designed. Additionally, an initial alignment algorithm using the data backtracking method is developed. In this algorithm, a rough initial alignment is conducted by the data from single-axis FOG, and a fine initial alignment is conducted by the data from FOG/MIMU. Finally, an experiment was conducted to validate this method. The experiment results indicate that the pitch and roll angle errors are less than 0.05°, and the azimuth angle errors are less than 0.2°. This improved the precision of the 3-D trajectory of underground pipelines.

## 1. Introduction

With the development of urban underground spaces, the pace of laying underground pipelines has accelerated. During the installation of new pipelines, accidents such as drilling through the pipe, frequently occur. Therefore, the trajectory of pre-existing underground pipelines must be obtained beforehand. Due to missing trajectory information for certain aged pipelines, measurement is required to determine their three-dimensional trajectories [1]. The inertial measurement method for pipelines is a novel pipeline measurement method based on the principles of inertial navigation [2]. Firstly, this method requires obtaining the initial attitude of the pipeline measuring instrument through initial alignment, and secondly, using inertial navigation algorithms to compute the three-dimensional trajectory of the pipeline. The initial alignment precision of the pipeline measuring instrument directly impacts the measurement accuracy of the pipeline’s three-dimensional trajectory.

Currently, a common initial alignment method for pipeline measuring instruments involves using a magnetometer to measure geomagnetic field information to obtain the instrument’s initial attitude [3]. However, the interference of the magnetic field in underground pipelines is so strong that it overwhelms the geomagnetic information [4,5], thereby rendering the magnetometer unable to obtain geomagnetic information [6]. Consequently, in conditions such as in pipelines with strong interfering magnetic fields, initial alignment methods relying on magnetometers become ineffective, necessitating alternative approaches for achieving high-precision initial alignment of pipeline measuring instruments.

In the aerospace domain, the combination of the three-axis Fiber Optic Gyro (FOG) and a triaxial accelerometer is always utilized to achieve high-precision initial alignment of the aircraft [7]. This method utilizes the three-axis FOG to measure Earth’s rotational angular velocity components [8,9], combined with gravity field components measured by the accelerometer, to solve for the initial attitude. Han et al. have proposed a model of the multiple stochastic errors in FOG which is used in the initial alignment of a self-designed FOG inertial navigation system (INS). Experimental results show that this method can further improve the alignment accuracy [10]. Li et al. have proposed a fast calibration method for a rotating inertial navigation system using an invariant extended Kalman filter. The fast calibration under a large error angle is realized [11], however, the three-axis FOG is so great that it cannot be applied in narrow pipelines and its high cost fails to meet the economic benefits of pipe measurement.

To address the issue of the large volume of the three-axis FOGs, some scholars have proposed employing a combination of a single-axis FOG and a Micro-Electro-Mechanical Systems Inertial Measurement Unit (MIMU) for initial alignment purposes [12,13]. Cai et al. have proposed an inertial measurement system composed of a skew single-axis FOG and a triaxial MEMS. Subsequently, they develop an initial alignment algorithm that uses three-position data backtracking [8]. However, it requires an accurate position provided by a turntable, making it unsuitable for pipe measurement applications. Tian et al. have proposed a novel FOG/MIMU hybrid system consisting of a triaxial MEMS and a slanted single-axis FOG [14]. This method utilizes the slanted single-axis FOG to enhance the accuracy of the MEMS gyroscope, thereby improving navigation precision. However, the alignment process necessitates precise rotation speeds, which cannot be provided in a pipeline, rendering this approach inapplicable in pipeline environments.

In response to the issue of initial alignment being unable to be completed in underground pipelines, a novel multi-position initial alignment method based on data backtracking for a single-axis FOG and MIMU is proposed. Firstly, the configuration of the sensors is determined. Secondly, according to the three-point support structure of the pipeline measuring instrument, a three-position alignment scheme is designed. Furthermore, an initial alignment algorithm using the data backtracking method is developed, commencing with the use of single-axis FOG/MIMU measurement for analytical backtracking coarse alignment to acquire a rough initial attitude, followed by the employment of MIMU measurement for backtracking fine alignment to attain high-precision initial orientation in a short period. This study presents a novel methodology for high-precision detection of underground pipelines.

The remaining structure of this study is as follows: Section 2 determines the configuration scheme for the single-axis FOG/MIMU composite sensor, designs a three-position initial alignment plan, and develops a three-position data backtracking-based initial alignment algorithm. Section 3 verifies the accuracy of the algorithm via simulation and experimentation. Section 4 provides a discussion. Section 5 concludes the study.

## 2. A Multi-Position Backtracking Alignment Method Based on Single-Axis FOG/MIMU

### 2.1. Sensor Configuration Scheme for Single-Axis FOG/MIMU

**Figure 1:**
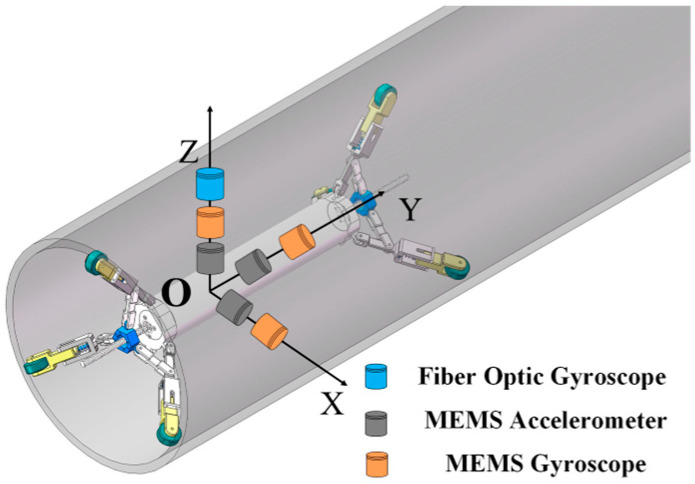
Structure of pipeline measuring instrument.

**Figure 2:**
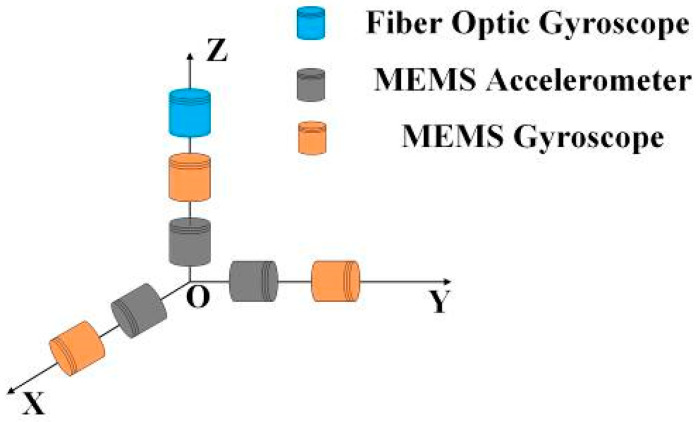
Sensor installation structure.

The structure of the pipeline measuring instrument is depicted in Figure 1. The pipeline measuring instrument features a three-point support structure at both its front and rear, ensuring that it remains centered in the pipeline throughout the measurement process. Based on the three-point support structure of the measuring instrument, the sensor configuration scheme is established, as illustrated in Figure 2. With O serving as the origin, a body-fixed coordinate system b is established, where OY points in the direction of the instrument’s forward motion, OX extends perpendicularly to OY towards the right, and OZ rises vertically above the XOY plane. The MEMS gyroscope and MEMS accelerometer are mounted along the OX, OY, and OZ axes, with the single-axis FOG installed vertically upward along the OZ axis.

### 2.2. Multi-Position Initial Alignment Process

**Figure 3:**
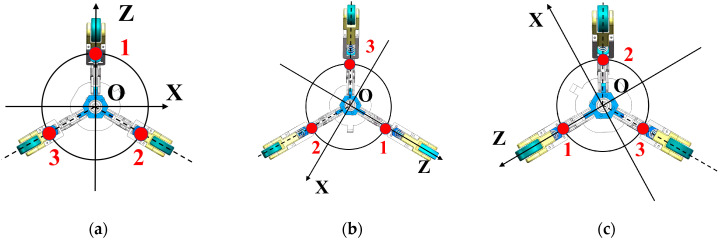
Initial alignment process (**a**) Position 1 (**b**) Position 2 (**c**) Position 3.

The multi-position initial alignment necessitates providing rotation angles between multiple positions, which is unavailable in the pipeline. Based on the three-point support structure of the pipe measuring instrument, the angle between each pair of support wheels is precisely 120°, which can furnish relatively accurate rotation angles. Thus, the three-position alignment method is adopted, wherein the measuring instrument is rotated in the pipeline, causing each of the three support wheels to point towards predetermined fixed orientations, thereby obtaining measurement at the three positions. The specific rotation process is illustrated in Figure 3.

**Figure 4:**
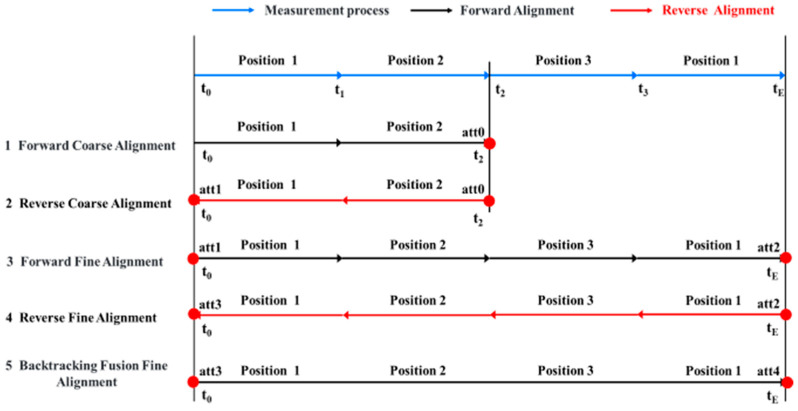
Data backtracking solution process.

After the measurement is completed, the data processing workflow is as follows in Figure 4:

Firstly, the multi-sensor measurement is integrated in chronological order. Utilizing single-axis FOG measurement from Position 1 and Position 2 (t_0_–t_2_), the earth’s rotation velocity components along the OX, OY, and OZ axes are computed. The single-axis FOG measurement and the triaxial accelerometer measurement of MIMU are used for forward coarse alignment and acquired a rough initial attitude att0 at time t_2_.

Subsequently, the single-axis FOG and the triaxial accelerometer of MIMU measurement from Position 1 and Position 2 (t_2_–t_0_) are integrated in reverse chronological order. Employing the rough initial attitude data att0 at time t_2_, a backward coarse alignment is conducted, yielding a rough initial attitude att1 at time t_0_.

Thirdly, the single-axis FOG, the triaxial accelerometer, and the triaxial gyroscope of MIMU measurement (t_0_–t_E_) are integrated in forward chronological order. Employing the rough initial attitude att1 at time t_0_, a forward fine alignment is conducted, yielding a high-precision initial attitude att2 at time t_E_.

Next, the single-axis FOG, the triaxial accelerometer, and the triaxial gyroscope of MIMU measurement (t_E_–t_0_) are integrated in reverse chronological order. Employing the high-precision attitude att2 at time t_E_, a backward fine alignment is conducted, yielding a high-precision attitude att3 at time t_0_.

Finally, the single-axis FOG, the triaxial accelerometer, and the triaxial gyroscope of MIMU measurement are integrated in forward chronological order. Based on the high-precision attitude att3 at time t_0_, a higher-precision initial attitude att4 at time t_E_ is obtained through fine alignment, thereby completing the short-term high-precision initial alignment [15,16].

### 2.3. Multi-Position Initial Alignment Algorithm

**Figure 5:**
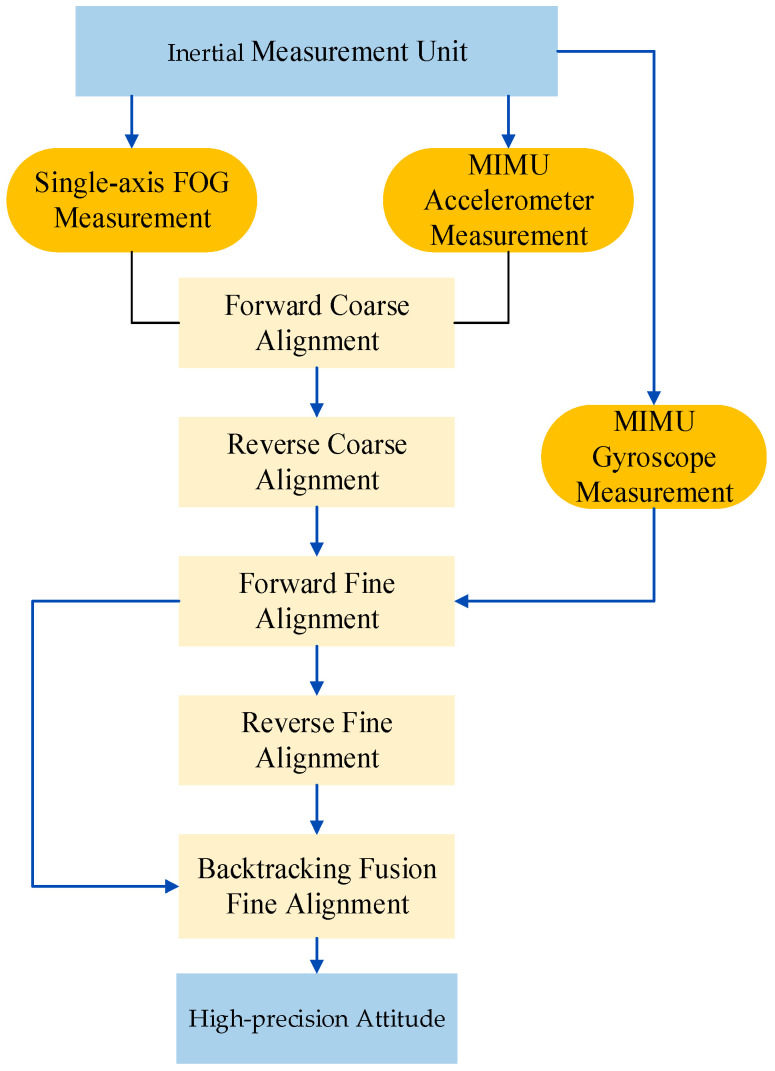
Data backtracking algorithm flow-chart.

The whole alignment algorithm included “Forward Coarse Alignment”, “Reverse Coarse Alignment”, “Forward Fine Alignment”, “Reverse Fine Alignment”, and “Backtracking Fusion Fine Alignment”. The initial attitude of high precision is obtained by calculating the measurement of single-axis FOG, MIMU accelerometer, and MIMU gyroscope. The whole alignment algorithm flow is as follows in Figure 5:

#### 2.3.1. Multi-Position Coarse Alignment Algorithm

First, an analytic coarse alignment is performed. The gravity vector $g^{n}$ and the earth’s rotational angular velocity vector $\omega_{ie}^{n}$ in the navigation coordinate system (East-North-Up) can be expressed as follows:

(1) $$g^{n}=\left[\begin{array}{c} 0\\ 0\\ -g \end{array}\right],$$

(2) $$\omega_{ie}^{n}=\left[\begin{array}{c} 0\\ \omega_{ie}\cos L\\ \omega_{ie}\sin L \end{array}\right]=\left[\begin{array}{c} 0\\ \omega_{N}\\ \omega_{U} \end{array}\right],$$

where L denotes the local latitude, g denotes the magnitude of the gravitational acceleration, $\omega_{ie}$ denotes the earth’s rotational angular velocity, and $\omega_{N}$ and $\omega_{U}$ are the northward and upward components of the earth’s rotational angular velocity.

Since the alignment process is static and the measurement instrument’s precision satisfies that sensor errors are much smaller than the measurement signal, angular velocity measurement in inertial navigation and the specific force equation can be represented as:

(3) $$C_{b}^{n}\omega_{ib}^{b}=\omega_{ie}^{n},$$

(4) $$C_{b}^{n}f_{sf}^{b}=-g^{n},$$

where matrix $C_{b}^{n}$ denotes the attitude matrix of the carrier system (b-system) relative to the navigation coordinate system (n-system), ${\vec{\omega }}_{ib}^{b}$ denotes the gyroscope output value, and ${\vec{f}}_{sf}^{b}$ is the specific force measured by the accelerometer.

According to the dual-vector attitude determination principle, selecting $-{\vec{g}}^{n}$ as the primary reference vector, the attitude matrix can be expressed as:

(5) $$C_{b}^{n}=\left[\begin{array}{c} -{\left(f_{sf}^{b}\times \omega_{ie}^{n}\right)}^{T}/\left|f_{sf}^{b}\times \omega_{ie}^{n}\right|\\ {\left(f_{sf}^{b}\times \omega_{ie}^{n}\times f_{sf}^{b}\right)}^{T}/\left|f_{sf}^{b}\times \omega_{ie}^{n}\times f_{sf}^{b}\right|\\ {\left(f_{sf}^{b}\right)}^{T}/\left|f_{sf}^{b}\right| \end{array}\right],$$

The attitude matrix obtained by solving can be used to calculate the attitude angles.

The analytic coarse alignment process requires the earth’s rotational angular velocity components $\omega_{ib}^{x}$, $\omega_{ib}^{y}$, and $\omega_{ib}^{z}$ in the OX, OY, and OZ directions of the body-fixed coordinate system b. However, in this study, the available sensor combination consists only of single-axis FOG, which can only sense the earth’s rotational angular velocity component along their respective axes. Consequently, it is necessary to derive $\omega_{ib}^{x}$, $\omega_{ib}^{y}$, and $\omega_{ib}^{z}$ through the analysis and solution of multi-position measurement.

Given that the earth’s rotational angular velocity is constant at all points outside the poles and that the alignment process involves rotation about the Y axis, the component of the earth’s rotational angular velocity in the OY direction remains unchanged. Consequently, the vector sum of the earth’s rotational angular velocity components in the XOZ plane remains constant throughout the alignment process.

**Figure 6:**
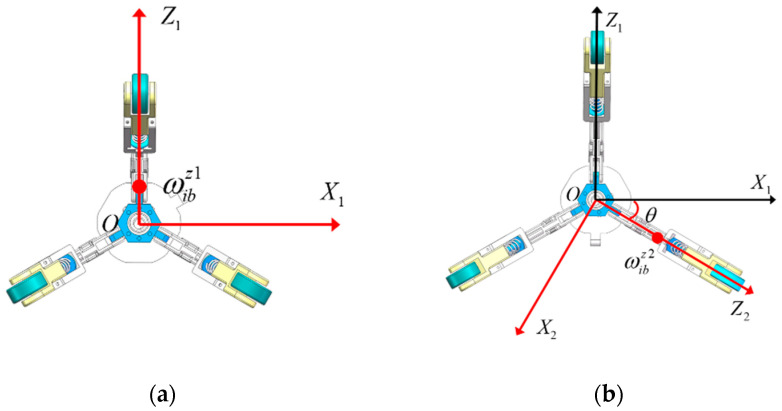
Single-axis FOG location (**a**) position 1 (**b**) position 2.

At position 1, a single-axis FOG measures the Earth’s rotation angular velocity component denoted as $\omega_{ib}^{z1}$. At position 2, a single-axis FOG measures the Earth’s rotation angular velocity component labeled as $\omega_{ib}^{z2}$, both depicted in Figure 6.

In the XOZ plane at position 1, there exists a vector $\omega_{ib}^{x1}$ in the OX direction perpendicular to $\omega_{ib}^{z1}$, such that the vector sum of $\omega_{ib}^{z1}$ and $\omega_{ib}^{x1}$ is the earth’s rotational angular velocity component in the XOZ plane. There exists a vector $\omega_{ib}^{x2}$ in the XOZ plane at position 2, perpendicular to $\omega_{ib}^{z2}$, such that the vector sum of $\omega_{ib}^{z2}$ and $\omega_{ib}^{x2}$ yields the component of Earth’s angular velocity in the XOZ plane.

Therefore, the components of $\omega_{ib}^{z2}$ and $\omega_{ib}^{x2}$ in the direction of $OZ^{\prime }$ at position 2 should be equal in magnitude to $\omega_{ib}^{z1}$. Utilize Formula (6) to calculate $\omega_{ib}^{x2}$.

(6) $$\omega_{ib}^{x2}=\frac{\omega_{ib}^{z1}+\omega_{ib}^{z2}\sin\theta }{\cos\theta },$$

Thereinto, $\theta =\frac{\pi }{6}$.

Similarly, the components of $\omega_{ib}^{z2}$ and $\omega_{ib}^{x2}$ in the direction of $OX^{\prime }$ should be equal in to $\omega_{ib}^{x1}$. Use Formula (7) to calculate $\omega_{ib}^{x1}$.

(7) $$\omega_{ib}^{x1}=\omega_{ib}^{x2}\sin\theta +\omega_{ib}^{z2}\cos\theta ,$$

Substitute (6) into (7) to obtain the result.

(8) $$\omega_{ib}^{x1}=\left(\omega_{ib}^{z1}+\omega_{ib}^{z2}\sin\theta \right)\tan\theta +\omega_{ib}^{z2}\cos\theta ,$$

Having obtained $\omega_{ib}^{x1}$, calculate magnitude $\omega_{ib}^{y1}$ using the vector addition formula to derive the earth’s angular velocity components $\omega_{ib}^{b}$ in the carrier coordinate system along the OX, OY, and OZ directions, as given by (9).

(9) $$\omega_{ib}^{y1}=\sqrt{{\left(\omega_{ib}\right)}^{2}-{\left(\omega_{ib}^{x1}\right)}^{2}-{\left(\omega_{ib}^{z1}\right)}^{2}}$$

Utilizing $\omega_{ib}^{b}$ and the accelerometer output values, solve to obtain the coarse initial attitude.

#### 2.3.2. Multi-Position Fine Alignment Algorithm

Following the coarse alignment, perform fine alignment using a Kalman filter [17].

The state equation and measurement equation for the Kalman filter are set as follows:

(10) $$\left.\begin{array}{c} \dot{X}=FX+W\\ Z=HX+V \end{array}\right\},$$

Thereinto,

$$X={\left[\phi E\;\phi N\;\phi U\;\delta v_{E}\;\delta v_{N}\;\delta v_{U}\;\varepsilon_{x}^{b}\;\varepsilon_{y}^{b}\;\varepsilon_{z}^{b}\;\nabla_{x}^{b}\;\nabla_{y}^{b}\;\nabla_{z}^{b}\right]}^{T},$$

$$F=\left[\begin{array}{c} \begin{array}{cccc} -(\omega_{ie}^{n}\times ) & 0_{3\times 3} & -C_{b}^{n} & 0_{3\times 3} \end{array}\\ \begin{array}{cccc} -(g^{n}\times ) & 0_{3\times 3} & 0_{3\times 3} & C_{b}^{n} \end{array}\\ 0_{6\times 12} \end{array}\right],$$

$$W^{b}={\left[\begin{array}{cccccc} \omega_{gx}^{b} & \omega_{gy}^{b} & \omega_{gz}^{b} & \omega_{ax}^{b} & \omega_{ay}^{b} & \omega_{az}^{b} \end{array}\right]}^{T},$$

$$Z={\left[\begin{array}{ccc} \delta v_{E} & \delta v_{N} & \delta v_{U} \end{array}\right]}^{T},$$

$$H=\left[\begin{array}{ccc} 0_{3\times 3} & I_{3\times 3} & 0_{3\times 6} \end{array}\right],$$

$$V={\left[\begin{array}{ccc} V_{E} & V_{N} & V_{U} \end{array}\right]}^{T},$$

where ϕE, ϕN, and ϕU denote the attitude angle errors in the east, north, and up directions; $\delta v_{E}$, $\delta v_{N}$, and $\delta v_{U}$ denote the velocity errors in the east, north, and up directions; $\varepsilon_{x}^{b}$, $\varepsilon_{y}^{b}$, and $\varepsilon_{z}^{b}$ are the random drift errors of the gyroscopes; $\nabla_{x}^{b}$, $\nabla_{y}^{b}$, and $\nabla_{z}^{b}$ are the constant biases of the accelerometers; $\omega_{gx}^{b}$, $\omega_{gy}^{b}$, and $\omega_{gz}^{b}$ are the equivalent gyro noise; $\omega_{ax}^{b}$, $\omega_{ay}^{b}$, and $\omega_{az}^{b}$ are the equivalent accelerometer noise; $V_{E}$, $V_{N}$, and $V_{U}$ are the equivalent eastward, northward, and upward velocity measurement noises, respectively.

#### 2.3.3. Multi-Position Backtracking Alignment Algorithm

To achieve high-precision initial alignment, two rounds of reverse data are employed to enhance alignment accuracy. Firstly, after completing forward coarse alignment, to extend the time for forward fine alignment and thereby improve alignment precision, a reverse coarse alignment is performed for the first time, yielding rough attitude information at the measurement starting time. After completing the forward fine alignment, to further enhance the alignment precision, a second reverse fine alignment is conducted, resulting in highly accurate initial attitude information.

The reverse initial alignment and the reverse navigation process employ the same reverse data methods [18].

- Forward navigation formula

The solution formula for the forward navigation algorithm can be expressed as:

(11) $$C_{b,k}^{n}=C_{b,k-1}^{n}(I+T_{s}\omega_{nb,k}^{b}\times ),$$

(12) $$V_{k}^{n}=V_{k-1}^{n}+T_{s}[C_{b,k-1}^{n}f_{k-1}^{b}-(2\omega_{ie,k-1}^{n}+\omega_{en,k-1}^{b})\times V_{k-1}^{n}+g^{n}],$$

(13) $$L_{k}=L_{k-1}+\frac{T_{s}V_{N,k-1}^{n}}{R_{M}+h_{k-1}},$$

(14) $$\lambda_{k}=\lambda_{k-1}+\frac{T_{s}V_{E,k-1}^{n}\sec L_{k-1}}{R_{N}+h_{k-1}},$$

(15) $$h_{k}=h_{k-1}+T_{s}V_{U,k-1}^{n},$$

Thereinto,

$$\begin{array}{l} \omega_{nb}^{b}=\omega_{ib}^{b}-{\left(C_{b}^{n}\right)}^{T}(\omega_{ie}^{n}+\omega_{en}^{n})\\ \omega_{en}^{n}={\left[-\frac{V_{N}^{n}}{R},\frac{V_{E}^{n}}{R},\frac{V_{E}^{n}\tan L}{R}\right]}^{T}\\ \omega_{ie}^{n}={\left[0,\omega_{ie}\cos L,\omega_{ie}\sin L\right]}^{T}\\ g^{n}=[0;0;-g] \end{array}$$

in the above formula, the superscript n represents the *ENU* system, while the superscript b represents the carrier coordinate system. The subscript k denotes the forward time sequence, with k increasing from 1 to N. $T_{S}$ is the sampling interval, $V^{n}$, L, λ, and h represent the velocity, latitude, longitude, and altitude, respectively. $R_{M}$ and $R_{N}$ are the meridian and equator radii of the local Earth, respectively. (•×) is the antisymmetric matrix by the vector •, and k is the forward sampling sequence, with k increasing from 1 to N.

b. Reverse navigation formula

The reverse navigation algorithm can be represented as:

(16) $${\hat{C}}_{b,p}^{n}={\hat{C}}_{b,p-1}^{n}(I+T_{s}{\hat{\omega }}_{nb,k-1}^{b}\times ),$$

(17) $${\hat{V}}_{p}^{n}={\hat{V}}_{p-1}^{n}+T_{s}[{\hat{C}}_{b,p-1}^{n}{\hat{f}}_{sf,p}^{b}-(2{\hat{\omega }}_{ie,p-1}^{n}+\omega_{en,p-1}^{b})\times {\hat{V}}_{p-1}^{n}+g^{n}],$$

(18) $${\hat{L}}_{p}={\hat{L}}_{p-1}+\frac{T_{s}{\hat{V}}_{N,p-1}^{n}}{R_{M}+{\hat{h}}_{p-1}},$$

(19) $${\hat{\lambda }}_{p}={\hat{\lambda }}_{p-1}+\frac{T_{s}{\hat{V}}_{E,p-1}^{n}\sec{\hat{L}}_{p-1}}{R_{N}+{\hat{h}}_{p-1}},$$

(20) $${\hat{h}}_{p}={\hat{h}}_{p-1}+T_{s}{\hat{V}}_{U,p-1}^{n},$$

Thereinto, ${\tilde{\omega }}_{nb,k-1}^{b}=-[\omega_{ib,k-1}^{b}-{\left(C_{b,k}^{n}\right)}^{T}(\omega_{ie,k}^{n}+\omega_{en,k}^{n})]$.

In the above formula, the subscript k denotes the time sequence in the forward direction, with p decreasing from N to 1.

At any moment, the attitudes and positions obtained through forward and reverse calculations are identical, with the only distinction being the opposite directions of the velocity vectors. By taking the gyroscope sample outputs, the Earth’s rotation angular velocity, and the opposite direction of the velocity, reverse calculations are achieved.

This study realizes the optimal estimation of attitude angles through the combination of multiple iterations of forward and reverse initial alignments. This method enhances the overall precision and reliability of short-time initial alignment.

## 3. Experiment

### 3.1. Simulation Experiment

#### 3.1.1. Simulation Experiment Conditions

In the simulation experiment, the bias of the single-axis fiber optic gyroscope is set to 0.1 deg/h with a random walk noise of 0.01 $\deg/\sqrt{h}$; the bias of the MEMS gyroscope is set to 4 deg/h and the random walk noise is set to 0.1 $\deg/\sqrt{h}$; whereas, the bias of the MEMS accelerometer is configured at 15 μg, accompanied by a random walk noise of 10 μg/h. The initial pitch angle is set to 0°, the roll angle to 0°, and the azimuth angle to 60°. A three-position alignment procedure is adopted, with the system remaining stationary for 180 s at each position.

#### 3.1.2. Simulation Experiment Results Analysis

**Figure 7:**
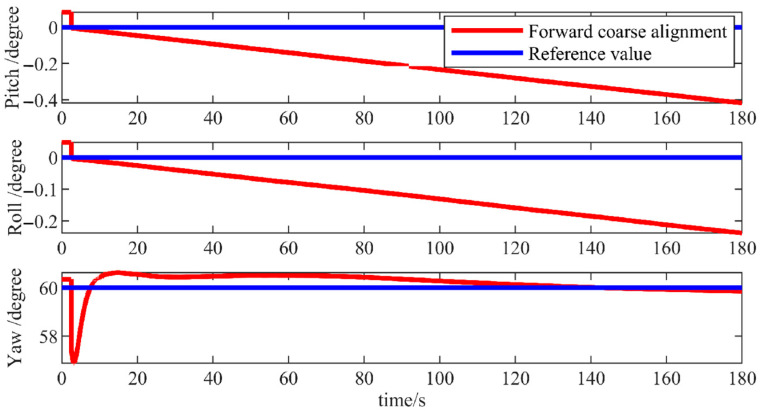
Forward rough alignment simulation results.

Firstly, a forward coarse alignment data resolution was conducted. As depicted in Figure 7, pitch and roll angle errors were approximately 0.5° while the error in azimuth angle was around 0.5°, generating a rough initial attitude estimation. Further alignment was required to enhance the accuracy of the alignment.

**Figure 8:**
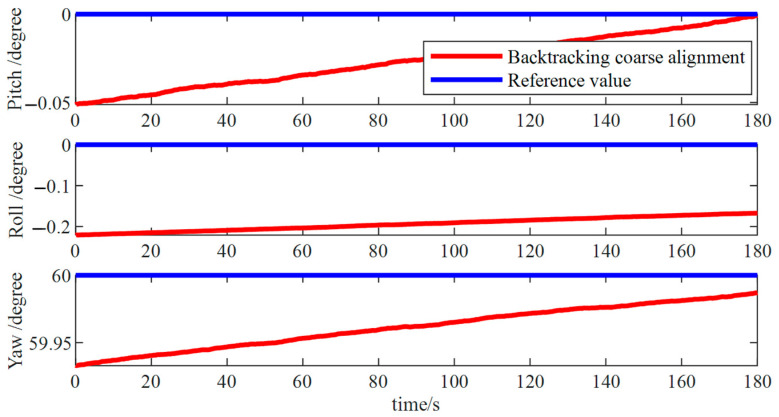
Reverse rough alignment simulation results.

Using the outcome from the forward coarse alignment, a reverse coarse alignment was performed to obtain the attitude information at the initial measurement instance (Figure 8). During the reverse coarse alignment phase, pitch and roll angle errors were approximately 0.05°, and the error in azimuth angle was about 0.05°, indicating an improvement over the forward coarse alignment results. However, these errors exhibited drift over time. Fine alignment was required to enhance the stability of the attitude determination.

**Figure 9:**
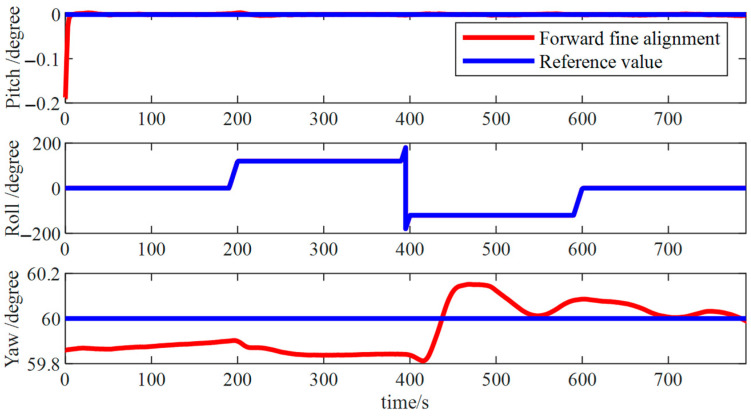
Forward fine alignment simulation results.

Figure 9 showed pitch and roll angle errors were approximately 0.01° and an azimuth angle error of around 0.2°, marking a notable increase in both precision and stability compared to previous stages. However, an error persisted in the initial attitude estimation, necessitating a reverse fine alignment process to attain highly accurate initial attitude information.

**Figure 10:**
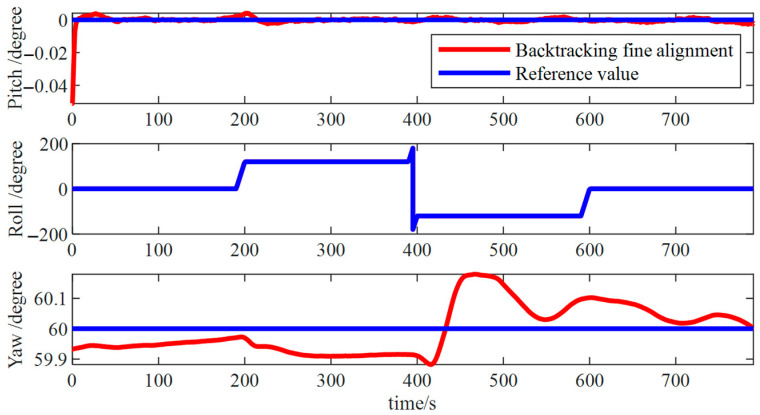
Backtracking fine alignment simulation results.

**Figure 11:**
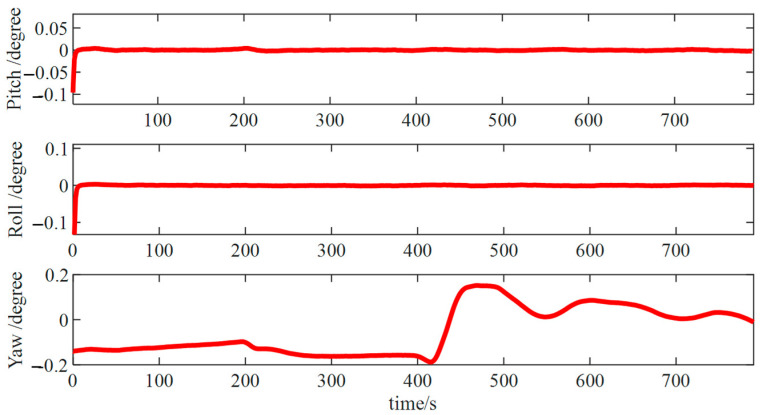
Backtracking fine alignment error.

Finally, the backtrack fine alignment was executed (Figure 10), yielding errors (Figure 11) in pitch and roll angles of about 0.01° and an azimuth angle error of roughly 0.05°. These accuracies met the alignment precision requirements, thereby the accuracy of the algorithm was verified.

### 3.2. Pipeline Experiment

#### 3.2.1. Experiment Conditions

**Figure 12:**
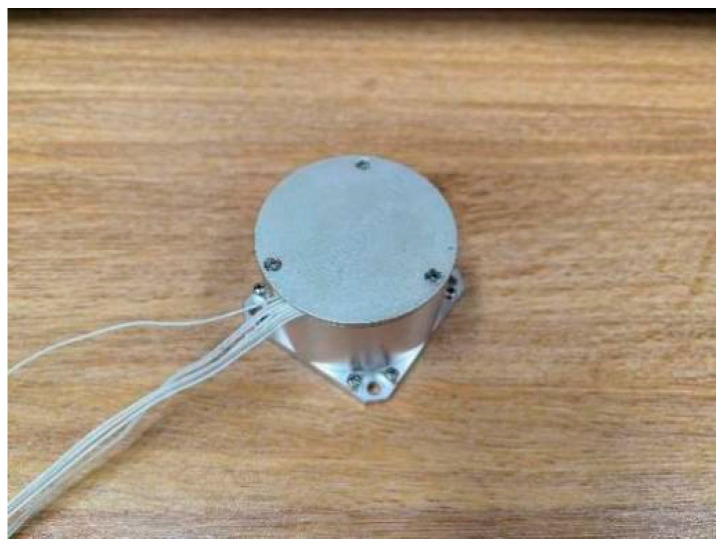
XD-50-FOG fiber optic gyroscope.

**Figure 13:**
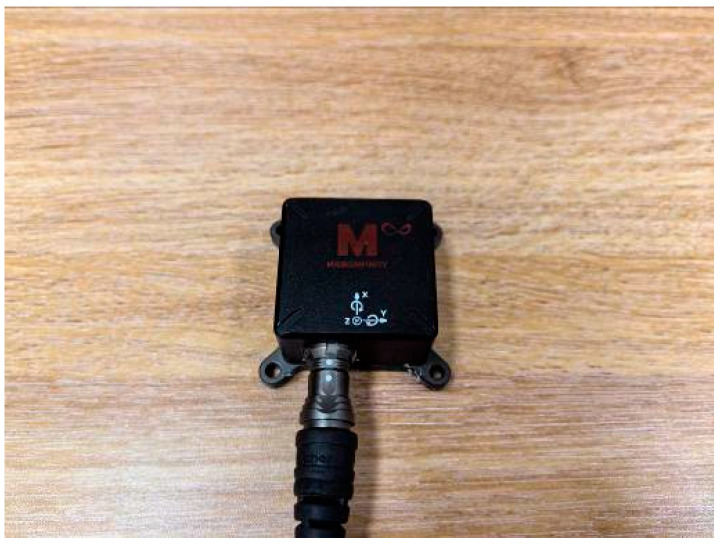
XNS100C sensor.

To further corroborate the algorithm’s accuracy, experiments were conducted using the single-axis fiber optic gyroscope XD-50-FOG (Luoyang new channel, Luoyang, China) (Figure 12) and the three-axis MEMS sensor XNS100C (MICROINFINITY, Suwon, Republic of Korea) (Figure 13). The high-precision fiber optic gyroscope inertial navigation system PA-GS300’s resolved values served as reference data. A three-axis turntable was employed for rotational experimentation.

The bias instability of XD-50-FOG was 0.5 deg/h and the walk randomly of XD-50-FOG was 0.2 $\deg/\sqrt{h}$. Parameters of XNS100C included bias instability of the accelerometer was 0.1 mg, walk randomly of the accelerometer was 340 μg/HZ, bias instability of the gyroscope was 12 deg/h, and walk randomly of the gyroscope was 0.2 $\deg/\sqrt{h}$. The attitude angle error of PA-GS300 was 0.05 deg/h and the heading hold accuracy was 0.3 deg/h.

**Figure 14:**
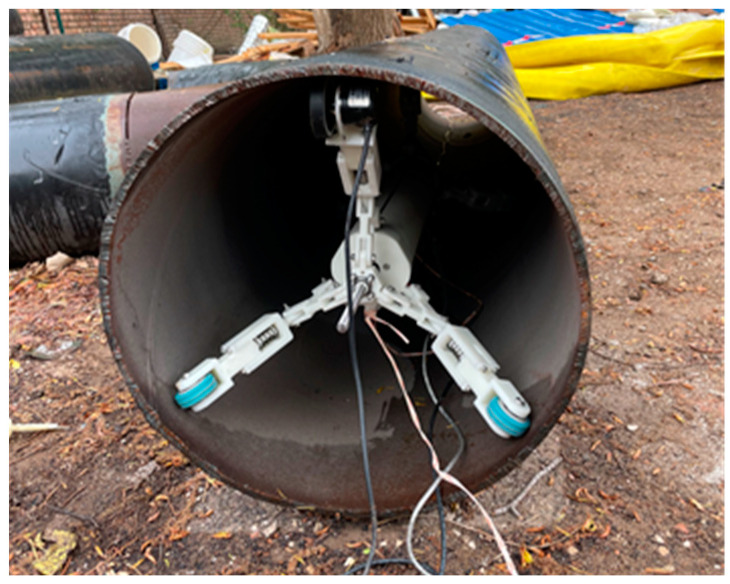
Sensor installation diagram.

The single-axis FOG and MIMU was installed in the pipeline measuring instrument and placed pipeline measuring instrument inside the pipeline. The pipeline measuring instrument was kept level, the measured roll and pitch was approximately 0°, and the azimuth was approximately −55° (Figure 14).

The alignment procedure was as follows:

1. The sensors are powered on, and measurement acquisition is initiated.
2. The system is left stationary for 3 min.
3. The system is rotated 120 degrees clockwise by motor rotation.
4. The system is left stationary for 3 min.
5. The system is rotated 120 degrees clockwise by motor rotation.
6. The system is left stationary for 3 min.
7. The system is rotated 120 degrees clockwise by motor rotation.
8. The system is left stationary for 3 min.

In total, the entire alignment process took approximately 12 min.

#### 3.2.2. Experiment Results Analysis

**Figure 15:**
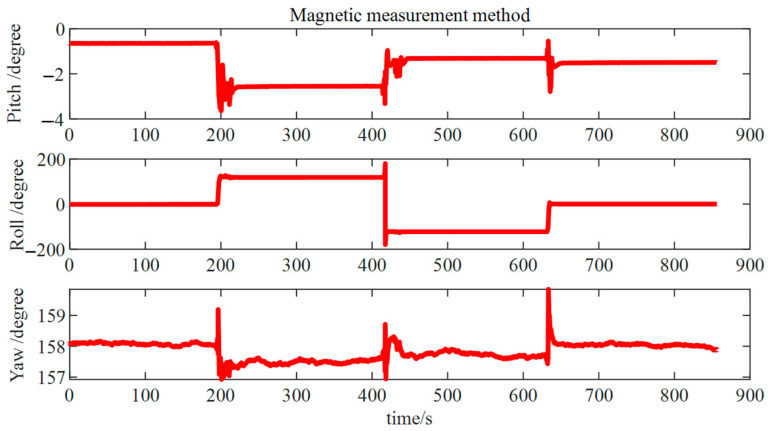
Magnetic measurement method results.

Figure 15 shows the result of the traditional measurement method. We can observe that the pitch and roll variation trends of the two figures match the true value. However, due to the interference of the magnetic field in the metal pipe, the result of the magnetic measurement method deviates from the real value.

**Figure 16:**
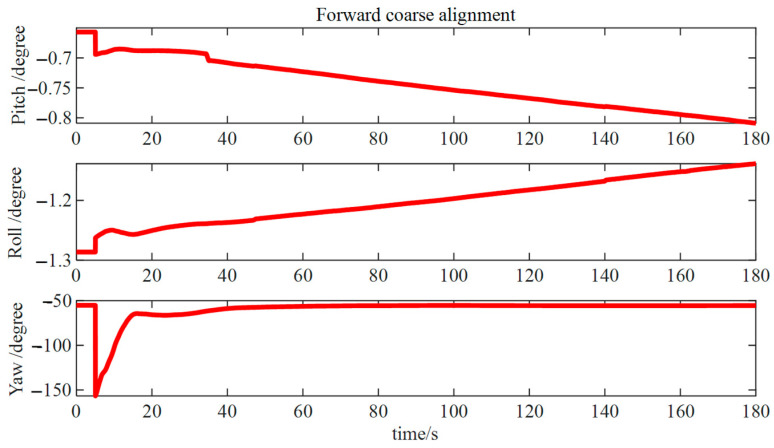
Forward rough alignment results.

In Figure 16, it can be observed that after performing 180 s of forward coarse alignment, the computed results deviate from the reference values by approximately 0.1° for both pitch and roll angles and approximately 1° for the azimuth angle, acquiring a rough initial attitude estimation. Further alignment was required to enhance the accuracy of the alignment.

**Figure 17:**
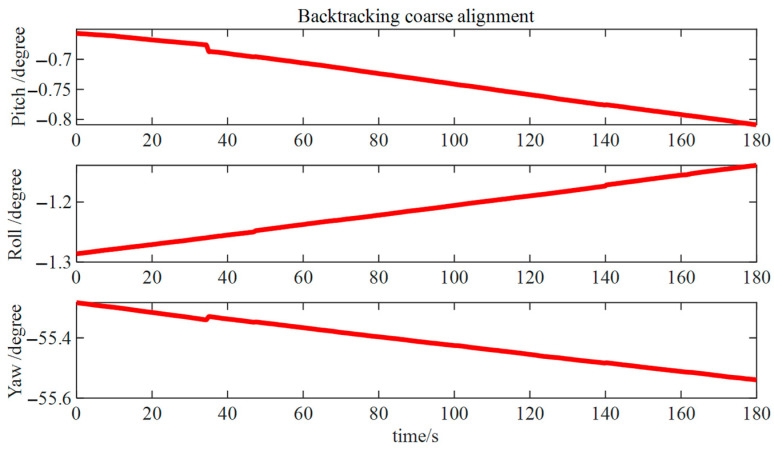
Reverse rough alignment results.

Following the completion of the forward coarse alignment, a reverse coarse alignment was conducted to acquire rough attitude data at the onset of measurements. Figure 17 shows that the errors in pitch and roll angles are roughly 0.1°, while the error in azimuth angle was approximately 0.5°. The larger error in the azimuth angle required fine alignment to enhance the overall alignment accuracy.

**Figure 18:**
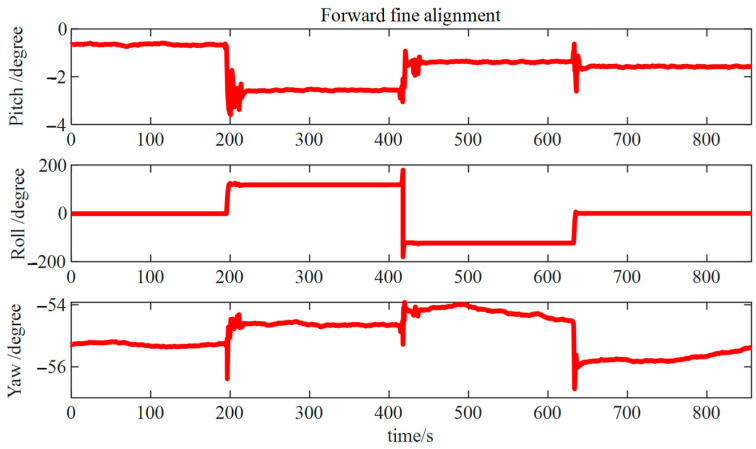
Forward fine alignment results.

As illustrated in Figure 18, in the forward fine alignment calculation process, pitch and roll angle errors were approximately 0.2°, whereas the error in the azimuth angle remained approximately 1°. As evident in the figure, errors existed in the initial attitude, requiring a backtrack fine alignment process to attain highly accurate initial attitude information. 

**Figure 19:**
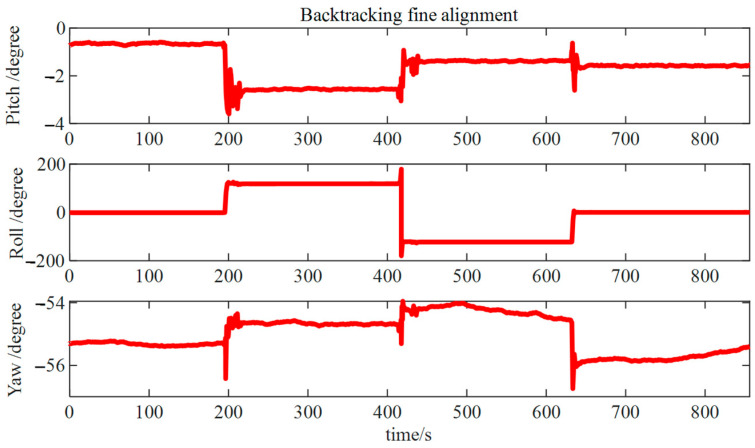
Backtracking fine alignment results.

The results from the backtracking fine alignment (Figure 19) indicate that pitch and roll angle errors were approximately 0.05°, while the error in the azimuth angle was approximately 0.2°.

## 4. Discussion

This study proposes a multi-position data backtracking initial alignment method based on a MIMU/FOG composite sensor system. The main advantages of this method include: (1) Method is not affected by magnetic interference, which has broad application scenarios. (2) High-precision initial alignment is achieved in a short period by reusing data. (3) It meets the economic efficiency of measurement while ensuring high-precision measurements. However, there remains room for further optimization:

1. The calculation results during the rotation phase exhibit relatively large errors. The calculation results from the MEMS sensors show a notable offset occurring during the rotation phase, with substantial discrepancies compared to the control group. The analysis suggests that the discrepancy may stem from MEMS sensors exhibiting different error characteristics under static and dynamic conditions. The calibration process has only accounted for static errors, failing to address the dynamic errors adequately. During the rotation phase, substantial measurement errors lead to deviations in the calculated results. Future work will endeavor to optimize the algorithm and estimate and suppress dynamic errors during the rotation phase, further enhancing the alignment accuracy.

2. The repeatability of random drift of the MEMS gyroscope is poor. In the calculation output values from MEMS sensors, it has been observed that the MEMS gyroscope exhibits poor repeatability of its random drift. To prevent significant azimuth angle shifts during fine alignment procedures, it is necessary to estimate and restrain the random drift of the MEMS gyroscope before each experimental run. In the condition where enhancement of sensor precision is unattainable, future research will focus on addressing how to restrain the impact of MEMS gyroscope random drift on initial attitude estimation.

3. Optimization of the experimental scheme can be achieved. The alignment scheme described in the text starts from an initial position, performing three clockwise rotations of 120 degrees each, pausing at each position, and returning to the starting point. To further enhance alignment accuracy, subsequent experimental designs will attempt clockwise rotations through three positions followed by counter-clockwise rotations along the same path, while reducing the duration of stationary periods. Under the condition that the total alignment duration remains constant, efforts will be made to increase alignment precision by calibrating the errors.

## 5. Conclusions

To achieve initial alignment in underground pipelines, a novel multi-position initial alignment method based on data backtracking for a single-axis FOG and a three-axis MIMU is proposed. Firstly, we proposed a three-point support measurement structure and sensor configuration scheme. Secondly, according to the three-point support structure of the pipeline measuring instrument, a three-position alignment scheme was designed. Additionally, we proposed an initial alignment algorithm using the data backtracking method. Finally, we carried out a simulation and experiment to validate this method. The simulation results showed that the pitch and roll angle errors were less than 0.01°, and the azimuth angle errors were less than 0.05°. The experiment results indicated that the pitch and roll angle errors were less than 0.05°, and the azimuth angle errors were less than 0.2°. The results verified the accuracy of the algorithm. Consequently, the multi-position inertial alignment method proposed in this study enabled realized initial alignment under magnetically disturbed conditions, such as underground pipelines. This method has a solid application prospect in the case of magnetic field interference in underground pipelines and presents a new method for measuring the three-dimensional trajectory of underground pipelines. Subsequent research will further improve the alignment stability and shorten the alignment time by optimizing the experimental and data processing processes.

## Data Availability

The original contributions presented in this study are included in the article.
